# *Terminalia bellirica* Extract Inhibits Low-Density Lipoprotein Oxidation and Macrophage Inflammatory Response *in Vitro*

**DOI:** 10.3390/antiox5020020

**Published:** 2016-06-14

**Authors:** Miori Tanaka, Yoshimi Kishimoto, Emi Saita, Norie Suzuki-Sugihara, Tomoyasu Kamiya, Chie Taguchi, Kaoruko Iida, Kazuo Kondo

**Affiliations:** 1 Department of Food and Nutritional Sciences, Graduate School of Humanities and Sciences, Ochanomizu University, 2-1-1 Otsuka, Bunkyo-ku, Tokyo 112-8610, Japan; g1670502@edu.cc.ocha.ac.jp (M.T.); notch0708@gmail.com (N.S.-S.); iida.kaoruko@ocha.ac.jp (K.I.); 2 Endowed Research Department “Food for Health”, Ochanomizu University, 2-1-1 Otsuka, Bunkyo-ku, Tokyo 112-8610, Japan; saita.emi@ocha.ac.jp (E.S.); taguchi.chie@ocha.ac.jp (C.T.); kondo.kazuo@ocha.ac.jp (K.K.); 3 Research and Development Division, Toyo Shinyaku Co Ltd., 7-28 Yayoigaoka, Tosu-shi, Saga 841-0005, Japan; kamiyat@toyoshinyaku.co.jp; 4 Institute of Life Innovation Studies, Toyo University, 1-1-1 Izumino, Itakura-machi, Ora-gun, Gunma 374-0193, Japan

**Keywords:** *Terminalia bellirica*, polyphenol, lipid oxidation, macrophage, inflammation, atherosclerosis

## Abstract

The deciduous tree *Terminalia bellirica* found in Southeast Asia is extensively used in traditional Indian Ayurvedic medicine for the treatment of hypertension, rheumatism, and diabetes. The anti-atherogenic effect of *Terminalia bellirica* fruit has not been fully elucidated. Here, we investigated the effect of *Terminalia bellirica* extract (TBE) on low-density lipoprotein (LDL) oxidation and inflammation in macrophages. TBE showed 1,1-diphenyl-2-picrylhydrazyl (DPPH) radical scavenging activity (EC_50_: 7.2 ± 1.2 μg/mL) and 15-lipoxygenase inhibitory activity. TBE also significantly inhibited free radical-induced LDL oxidation compared to the solvent control *in vitro*. In THP-1 macrophages, TBE treatment resulted in significant decreases of the mRNA expression of tumor necrosis factor-alpha (TNF-α), interleukin-1beta (IL-1β), and lectin-like oxidized LDL receptor-1 (LOX-1). TBE also reduced matrix metalloproteinase (MMP)-9 secretion and intracellular reactive oxygen species (ROS) production in THP-1 macrophages. These results show that TBE has the inhibitory effects on LDL oxidation and macrophage inflammatory response *in vitro*, suggesting that its *in vivo* use might inhibit atherosclerosis plaque progression.

## 1. Introduction

*Terminalia bellirica* is a deciduous tree that is common in Southeast Asia. In traditional Indian Ayurvedic medicine, the fruit of *Terminalia bellirica* is extensively used as a folk medicine for the treatments of diabetes, hypertension, and rheumatism [1]. The major polyphenolic components of the fruit are reported to be gallic acid, ellagic acid, and chebulagic acid [2]. *Terminalia bellirica* extract (TBE), which is obtained from the fruit of *Terminalia bellirica*, has been reported to show hypolipidemic [3], hypoglycemic [4], and antihypertensive [5] properties in mice and in human subjects, and it is used as a dietary supplement.

Atherosclerosis, a major cause of death, is highly associated with the oxidative modification of low-density lipoprotein (LDL) and inflammation in the vascular wall. Oxidized LDL (oxLDL), which is detected in atherosclerotic lesions, is considered to be one of the main risk factors for atherosclerosis. Foam cell formation is mainly due to an uninterrupted uptake of oxLDL in macrophages, resulting in an excessive level of lipoprotein-derived cholesterol accumulation in the intima [6]. Scavenger receptors (e.g., class A scavenger receptor (SR-A), CD36, and lectin-like oxLDL receptor-1 (LOX-1)) are responsible for the uptake of modified LDL by macrophages [7]. In addition, oxLDL activates the cascade of local inflammation in the vascular wall by upregulating the expression of adhesion molecules, growth factors, and pro-inflammatory cytokines [8].

The macrophage-mediated inflammatory response plays a crucial role in the progression of atherosclerosis. In atherosclerotic lesions, macrophages release pro-inflammatory cytokines, reactive oxygen species (ROS), and matrix metalloproteinases (MMPs) [9,10]. Some reports have suggested that ROS may be involved in cell growth, adhesion, and vascular endothelial dysfunction [11,12,13]. ROS overproduction is associated with the production of many inflammatory mediators and a range of inflammation-related diseases [13]. MMPs, a family of Zn^2+^-dependent endopeptidases, have been linked to weakening of the vascular wall and plaque rupture due to degradation of the extracellular matrix [14,15]. The modulation of LDL oxidation and inflammation in macrophages would thus be important to therapeutic strategies against atherosclerosis. Dietary antioxidants such as polyphenols have been reported to prevent LDL oxidation [16,17] and inflammatory response in macrophages [18]. A negative correlation between polyphenol consumption and cardiovascular disease has been revealed by a number of epidemiologic studies [19,20,21].

TBE is known to contain polyphenols that have an antioxidant property. However, there is little information about the effects of TBE regarding the prevention of atherosclerosis. The aim of this study was to determine whether TBE has an antioxidant effect on LDL oxidation and whether it inhibits inflammatory mediator expression in macrophages.

## 2. Materials and Methods

### 2.1. Reagents

A hot water extract of *Terminalia bellirica* was provided by Toyo Shinyaku Co., Ltd. (Saga, Japan). The powder was dissolved in deionized water and used in the experiments. The contents of gallic acid and ellagic acid in the TBE stock solution (40 mg/mL) were 4.6 mg/mL and 0.16 mg/mL, respectively. RPMI-1640 medium, phorbol-12-myristate-13-acetate (PMA), Hank’s balanced salts solution (HBSS), and 2′,7′-dichlorofluorescein-diacetate (DCFH-DA) were purchased from Sigma-Aldrich (St. Louis, MO, USA). Fetal bovine serum (FBS) and penicillin/streptomycin were obtained from GIBCO (Life Technologies, Grand Island, NY, USA).

### 2.2. Determination of Total Polyphenol Content

The total polyphenol content was determined by a Folin–Ciocalteu assay, as described in [22]. In brief, the Folin–Ciocalteu phenol reagent (Nacalai Tesque, Kyoto, Japan) was added to the sample and incubated in 1.5% Na_2_CO_3_ solution for 2 h at 20 °C, and the absorbance was determined at 750 nm using a DU800 spectrophotometer (Beckman Coulter, Brea, CA, USA). The results are expressed as a (+)-catechin (Wako Pure Chemicals, Osaka, Japan) equivalent.

### 2.3. 1,1-Diphenyl-2-Picrylhydrazyl (DPPH) Radical Scavenging Activity

The free radical scavenging activity was determined using 1,1-diphenyl-2-picrylhydrazyl (DPPH) (Wako Pure Chemicals). An aqueous solution of TBE powder was mixed with 1 mL of 0.2 mM DPPH in ethanol. After incubation for 20 min at 37 °C, the absorbance of each solution was measured at 516 nm using the DU800 spectrophotometer. The concentration of TBE required to cause a 50% decrease in the absorbance at 516 nm relative to the control was then calculated.

### 2.4. Isolation of LDL from Human Subjects

Blood samples from fasting normolipidemic adult volunteers were collected in sodium EDTA-containing tubes. Plasma samples were immediately prepared by centrifugation at 3000 rpm for 15 min at 4 °C. The LDL was separated by single-spin density gradient ultracentrifugation at 100,000 rpm for 40 min at 4 °C using a TLA-100.4 fixed angle-rotor (Beckman Instruments, Fullerton, CA, USA). The LDL protein concentration was determined using a Micro BCA Protein Assay Kit (Pierce Laboratories, Rockford, IL, USA). All volunteers provided informed consent prior to sample collection. The study was conducted in accordance with the Declaration of Helsinki, and the protocol was approved by the Ethics Committee of Ochanomizu University.

### 2.5. LDL Lag Time Assay

We measured the LDL oxidizability as described in [23]. Briefly, the prepared LDL samples (final concentration of protein: 70 μg/mL) were oxidized with and without TBE by 400 μM 2,2-azobis-4-methoxy-2,4-dimethylvaleronitrile (V-70; AMVN-CH_3_O). We determined the kinetics of LDL oxidation by monitoring the absorbance of conjugated dienes at 234 nm, using the DU800 spectrophotometer at 4 min intervals at 37 °C.

### 2.6. 15-Lipoxygenase Inhibitory Activity

We tested the 15-lipoxygenase (15-LOX) inhibitory activity of TBE by using a Lipoxygenase Inhibitor Screening Assay Kit (Cayman Chemical, Ann Arbor, MI, USA). First, 90 μL of soybean 15-LOX enzyme solution and 10 μL of TBE solution were mixed in the testing wells. The reaction was initiated by adding 10 μL of arachidonic acid substrate solution to all of the wells. All of the testing wells were then placed on a shaker for 5 min, and 90 μL of chromogen was then added to each well to stop the enzyme catalysis and develop the reaction. The absorbance of hydroperoxides produced by 15-LOX from arachidonic acid was measured at 490 nm using a microplate reader (Bio Tek Instruments, Tokyo, Japan).

### 2.7. Cell Culture and Treatment

The human monocytic cell line THP-1 was obtained from the RIKEN Cell Bank (Ibaraki, Japan) and cultured in RPMI-1640 medium supplemented with 10% FBS, 100 U/mL penicillin, and 100 μg/mL streptomycin at 37 °C and 5% CO_2_. THP-1 cells were induced with 100 nM PMA for 48 h for differentiation into macrophages. THP-1 macrophages were treated with 25–100 μg/mL TBE for a maximum of 24 h. We confirmed that TBE did not affect cell viability under these conditions by MTT assay.

### 2.8. Real-Time RT-PCR

Total cellular RNA was extracted using the total RNA extract reagent RNAiso Plus (Takara Bio, Shiga, Japan). We reverse-transcribed first-stand complementary DNAs from 2 μg of the total RNA by using a High Capacity cDNA Reverse Transcription Kit (Applied Biosystems, Foster City, CA, USA). A real-time polymerase chain reaction (PCR) was performed on an ABI 7300 cycler (Applied Biosystems) with Power SYBR Green PCR mix (Applied Biosystems). The results are expressed as the copy number ratio of the target mRNAs to GAPDH mRNA. The primer sequences used for the analysis of tumor necrosis factor-alpha (TNF-α) (*TNF*), interleukin-1beta (IL-1β) (*IL1B*), class A scavenger receptor (SR-A) (*MSR1*), CD36 (*CD36*), and lectin-like oxidized LDL receptor-1 (LOX-1) (*OLR1*) are shown in Table 1.

### 2.9. Detection of MMP-9 Activity by Gelatin Zymography

We assessed the gelatinolytic activity of MMP-9 by performing a gelatin zymography assay, as described in [24]. First, 5 μL of culture supernatants were electrophoresed in 10% polyacrylamide gels containing 1% sodium dodecyl sulfate (SDS) and 0.1% gelatin. The gels were washed twice in 2.5% Triton X-100 for 30 min and incubated overnight at 37 °C in incubation buffer containing 50 mM Tris-HCl (pH 7.5), 150 mM NaCl, 10 mM CaCl_2_, and 1 nM ZnCl_2_. The gels were then stained in 0.25% Coomassie brilliant blue R-250 and destained using 35% ethanol and 10% acetic acid solution. Gelatinolytic activity was quantified by scanning densitometry with an ImageQuant LAS-4000 biomolecular imaging system (Fujifilm, Tokyo, Japan) and analyzed by Multi Gauge software, ver. 3.0 (Fujifilm).

### 2.10. Measurement of Intracellular ROS Production

We measured intracellular ROS production by using DCFH-DA as the fluorescent probe. THP-1 macrophages were incubated with 20 μM DCFH-DA/HBSS for 30 min, then treated with 25–100 μg/mL TBE in fresh HBSS for 2 h. The fluorescent intensity was detected at 485 nm excitation and 528 nm emission with the use of a microplate reader.

### 2.11. Statistical Analysis

All results are presented as mean ± standard deviation (SD). We performed a one-way analysis of variance (ANOVA) followed by Tukey’s *post hoc* test to compare treatment groups. Differences were considered significant when *p* < 0.05. We used the GraphPad Prism 5 software package (GraphPad Software, La Jolla, CA, USA) for all of the statistical analyses.

## 3. Results

### 3.1. The Total Polyphenol Content of TBE

The total polyphenol content of the TBE powder measured by the Folin–Ciocalteu assay was 231 ± 11 mg/g. According to the high-performance liquid chromatography (HPLC) analyses of the TBE stock solution conducted by the supplier (Toyo Shinyaku), the contents of gallic acid and ellagic acid were calculated to be 115 mg/g and 4 mg/g, respectively, suggesting that gallic acid is the major polyphenolic compound of TBE.

### 3.2. The DPPH Radical Scavenging Activity of TBE

**Figure 1:**
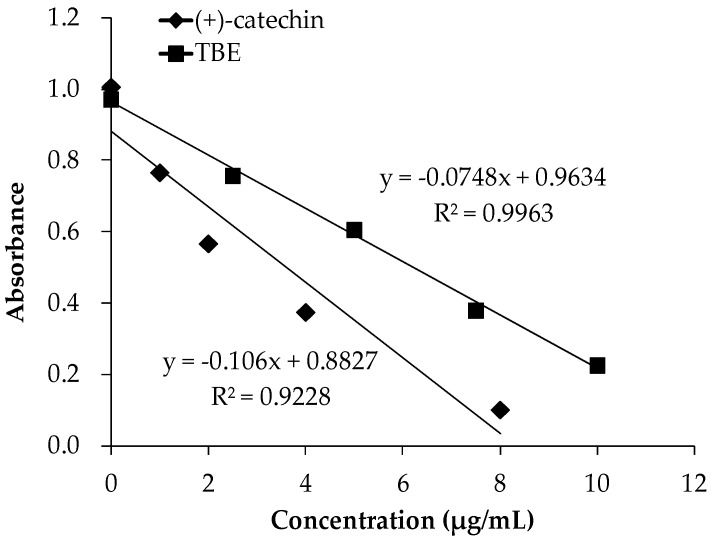
Decreases in the absorbance of 1,1-diphenyl-2-picrylhydrazyl (DPPH) radical at 516 nm, measured after 20 min at 37 °C, depending on the concentration of (+)-catechin and TBE solution.

The DPPH assay is based on the scavenging of the DPPH radical from the antioxidants, which produces a decrease in absorbance at 516 nm. The results of DPPH radical scavenging activity of TBE and the reference antioxidant (+)-catechin are presented in Figure 1. TBE showed DPPH radical scavenging activity: the EC_50_ value (the concentration reducing DPPH absorbance by 50%) was 7.2 ± 1.2 μg/mL.

### 3.3. TBE Slowed LDL Oxidation *In Vitro*

**Figure 2:**
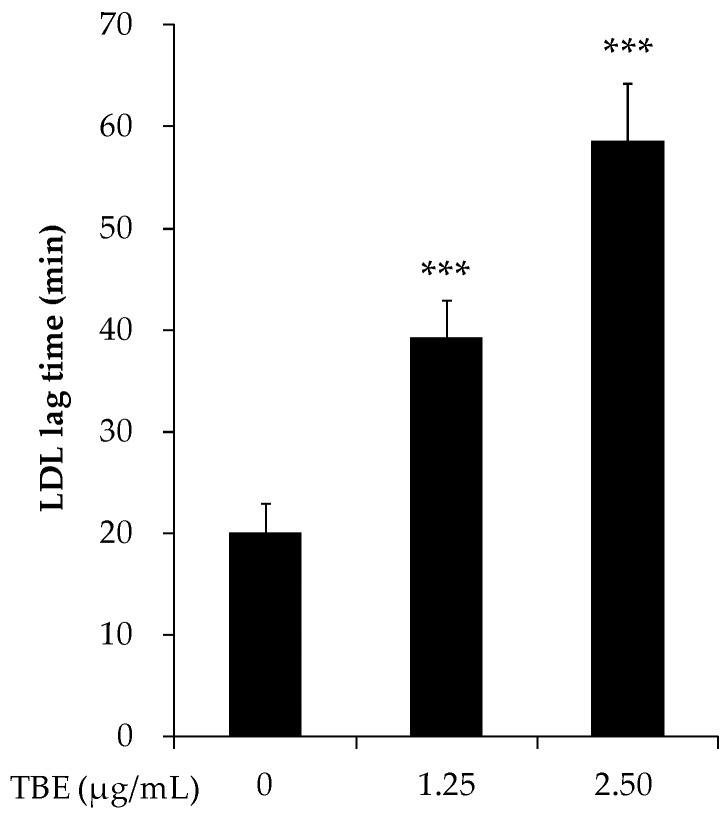
Effect of *Terminalia bellirica* extract (TBE) on low-density lipoprotein (LDL) oxidation lag time. LDL (70 μg/mL protein) was incubated with 400 μM of 2,2-azobis-4-methoxy-2,4-dimethylvaleronitrile (AMVN-CH_3_O) in the absence or presence of TBE at 37 °C. The lag time for LDL oxidation was defined as the time interval between the initiation and intercept of the 2 tangents drawn to the lag and propagation phase of the absorbance curve at 234 nm. Values are means ± SD (*n* = 5). *** *p* < 0.001, compared to untreated control group.

To evaluate the antioxidant effect of TBE on LDL oxidation, we performed an LDL lag time assay. As shown in Figure 2, TBE significantly prolonged the LDL oxidation lag time (1.25 μg/mL: 39.3 ± 3.7 min; 2.5 μg/mL: 58.6 ± 5.6 min; and control: 20.1 ± 2.9 min).

### 3.4. TBE Inhibited 15-Lipoxygenase (15-LOX) Activity

**Figure 3:**
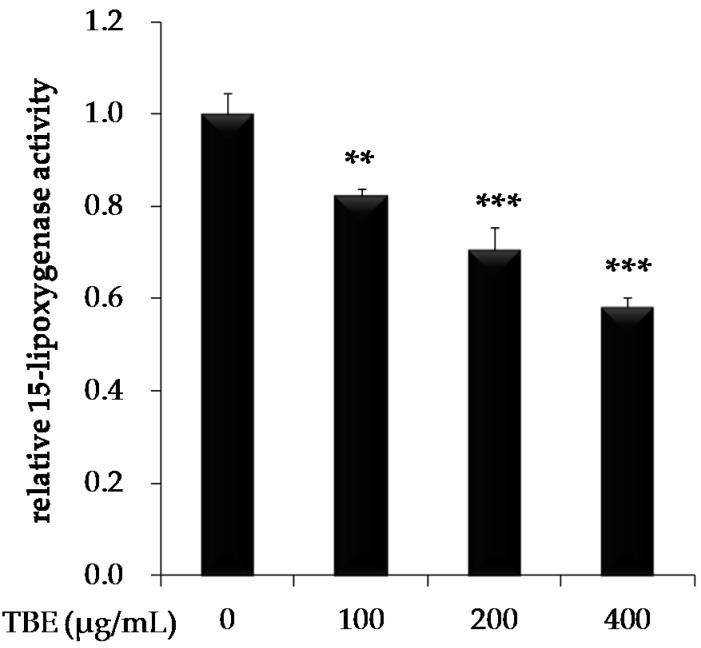
Effect of TBE on 15-lipoxygenase (15-LOX) activity. 15-LOX was mixed with arachidonic acid in the absence or presence of TBE. The absorbance of hydroperoxides produced by 15-LOX from arachidonic acid was measured at 490 nm. Values are means ± SD (*n* = 3). ** *p* < 0.01, *** *p* < 0.001, compared to untreated control group.

In order to test the ability of TBE to directly inhibit 15-LOX, a cell-free assay using soybean 15-LOX enzyme and arachidonic acid as the substrate was applied. TBE displayed 15-LOX inhibitory activity compared to the solvent control (100 μg/mL: 82.1% ± 1.7%; 200 μg/mL: 70.6% ± 4.8%; 400 μg/mL: 58.1% ± 2.2%) (Figure 3).

### 3.5. TBE Reduced the mRNA Expression of TNF-α and IL-1β in THP-1 Macrophages

**Figure 4:**
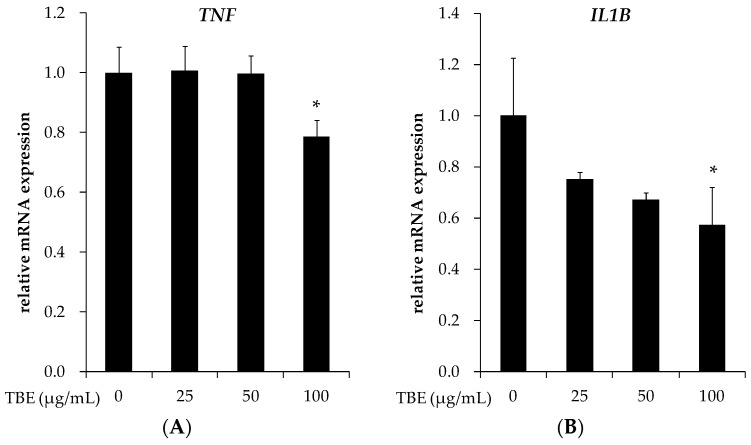
Effect of TBE on the mRNA expression of pro-inflammatory cytokines in macrophages. THP-1 macrophages were incubated with 25–100 μg/mL TBE for 24 h. *TNF* (**A**) and *IL1B* (**B**) mRNA expressions were measured by real-time RT-PCR. Values are means ± SD (*n* = 3). * *p* < 0.05, compared to untreated control group.

Since the expression of inflammatory cytokines is a well-known response to inflammation in macrophages, we examined the effects of TBE on the mRNA expression of TNF-α and IL-1β. Figure 4 shows that treatment with TBE (100 μg/mL) significantly reduced the mRNA expression of these genes.

### 3.6. TBE Suppressed the mRNA Expression of LOX-1 in THP-1 Macrophages

**Figure 5:**
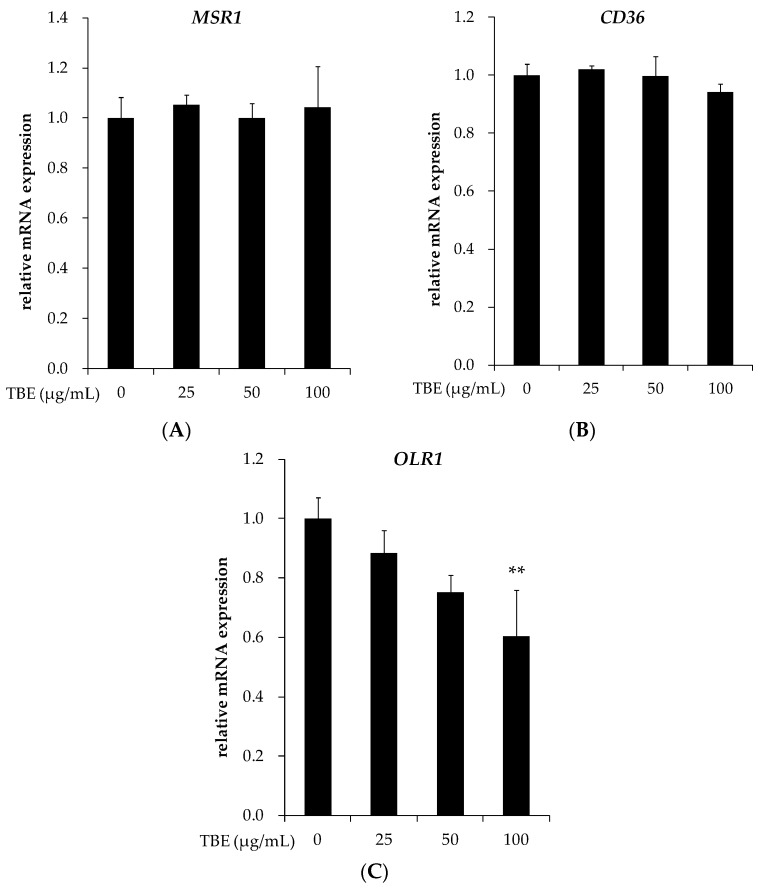
Effect of TBE on the mRNA expression of scavenger receptors in macrophages. THP-1 macrophages were incubated with 25–100 μg/mL TBE for 24 h. *MSR1* (**A**), *CD36* (**B**) and *OLR1* (**C**) mRNA expressions were measured by real-time RT-PCR. Values are means ± SD (*n* = 3). ** *p* < 0.01, compared to untreated control group.

It has been reported that SR-A, CD36, and LOX-1 play a critical role during lipid accumulation in macrophages. We examined the effects of TBE on the mRNA expression of these scavenger receptors. Incubation with TBE (100 μg/mL) significantly suppressed the mRNA expression of LOX-1 without affecting the expression of SR-A and CD36 (Figure 5).

### 3.7. TBE Reduced the MMP-9 Secretion in THP-1 Macrophages

**Figure 6:**
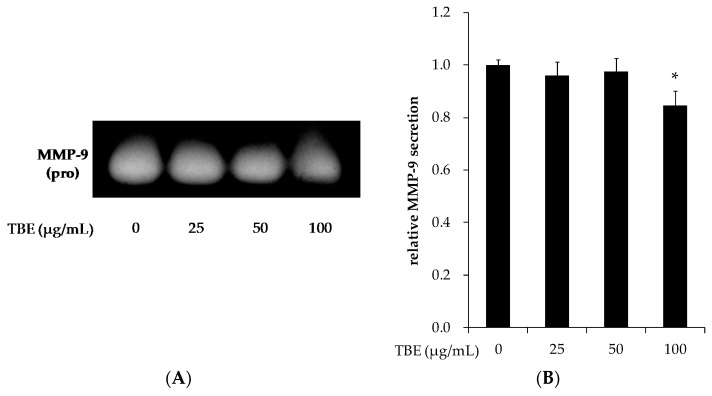
Effect of TBE on matrix metalloproteinase (MMP)-9 secretion in macrophages. THP-1 macrophages were incubated with 25–100 μg/mL TBE for 24 h. MMP-9 secretion in culture supernatants (**A**) and its quantification (**B**) were analyzed by gelatin zymography. Values are means ± SD (*n* = 3). * *p* < 0.05, compared to untreated control group.

MMP-9 induces atherosclerotic plaque rupture, and its expression is strongly correlated with lesion instability and the manifestation of atherosclerosis. To clarify the effect of TBE on MMP-9 activation in macrophages, we analyzed the secretion level of MMP-9 by conducting a gelatin zymography assay. Treatment with TBE (100 μg/mL) significantly reduced the MMP-9 secretion (Figure 6).

### 3.8. TBE Decreased the ROS Production in THP-1 Macrophages

**Figure 7:**
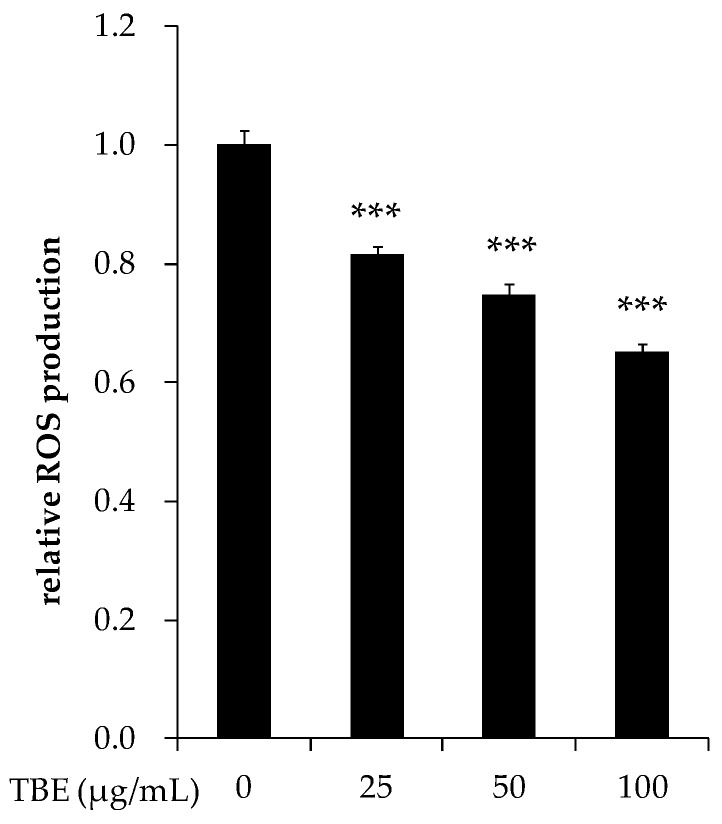
Effect of TBE on reactive oxygen species (ROS) production in macrophages. THP-1 macrophages were incubated with 25–100 μg/mL TBE for 2 h, and then intracellular ROS levels were measured by using 2′,7′-dichlorofluorescein-diacetate (DCFH-DA) (excitation 485 nm/emission 528 nm). Values are means ± SD (*n* = 3). *** *p* < 0.001, compared to untreated control group.

ROS play an important role in inflammatory mediator expression. To examine whether TBE exerted an anti-inflammatory effect by the downregulation of ROS production, we measured the intracellular ROS production in THP-1 macrophages by using DCFH-DA. As shown in Figure 7, incubation with TBE significantly decreased the ROS production in a dose-dependent manner.

## 4. Discussion

The oxidative modification of LDL plays an important role in the initiation and progression of atherosclerosis, inducing not only foam cell formation but also an inflammatory response. Lipid hydroperoxide and lysophosphatidylcholine, components of oxLDL, are known to increase the expression of adhesion molecules and growth factors, apoptosis, and the dysfunction of endothelial nitric oxide synthase in the vascular wall [25,26]. The release of pro-inflammatory cytokines and MMPs from macrophages is also promoted in the presence of oxLDL [27]. LDL oxidation results from various mechanisms, such as free radicals, active metal ions, and peroxidative enzymes including lipoxygenase and myeloperoxidase [28]. In the present study, we demonstrated that TBE contained polyphenols and showed DPPH radical scavenging activity. The fruit of *Terminalia bellirica* is known to contain several types of polyphenols (e.g., gallic acid, ellagic acid) [2]. Middha *et al.* [29] reported that gallic acid and ellagic acid had free radical scavenging activity and inhibited lipid peroxidation. In the present study, TBE significantly prolonged the LDL oxidation lag time, indicating the inhibition of the free radical-induced lipid peroxidation of LDL. We also observed that TBE displayed 15-lipoxygenase inhibitory activity. Some polyphenols (e.g., gallic acid, gallic acid glycoside) have been shown to inhibit lipoxygenase activity and lipoxygenase-mediated LDL lipid peroxidation [30,31]. These findings suggest that TBE may exert an antioxidant effect on LDL oxidation via the inhibition of both the radical reaction by free radicals and the non-radical reaction by peroxidative enzymes.

Macrophage-mediated inflammation is a well-known key step in atherosclerosis progression. Macrophages express scavenger receptors and secrete a number of cytokines and MMPs, which contribute to lesion progression, endothelial dysfunction, and plaque rupture. The inhibition of these inflammatory mediators would thus be useful for the control of inflammation. In the present study, TBE suppressed the expression of pro-inflammatory cytokines, LOX-1, MMP-9, and ROS production in THP-1 macrophages.

Pro-inflammatory cytokines such as TNF-α, IL-1β, and IL-6 are produced by activated macrophages and involved in the upregulation of the inflammatory response. TNF-α and IL-1β are considered key inflammatory cytokines associated with many inflammatory processes, including the expression of adhesion molecules and other cytokines [32]. We found that TBE reduced the mRNA expression of TNF-α and IL-1β in THP-1 macrophages. The deficiency of TNF and IL-1 has been shown to decrease the atherosclerotic lesion size in apoE^−/−^ mice [33,34].

Scavenger receptors are implicated in the uptake of oxLDL and foam cell formation in macrophages. Here, we showed that TBE suppressed the mRNA expression of LOX-1 without altering the expression of SR-A and CD36. LOX-1 is a major receptor for oxLDL, and its expression is enhanced in several disease states including diabetes, hypertension, and atherosclerosis [35]. Inoue *et al.* [36] demonstrated that the overexpression of LOX-1 induced foam cell accumulation and atherosclerotic lesion formation in apoE^−/−^ mice. In other studies, LOX-1 deletion resulted in a significant attenuation of the atherosclerosis plaque progression and inflammatory response [37,38]. The expression of LOX-1 is known to be upregulated by pro-inflammatory cytokines, oxLDL, and free radicals, suggesting its critical role for inflammation and the development of atherosclerosis [39,40]. In view of the function of LOX-1, the suppression of LOX-1 expression by TBE may contribute to the amelioration of foam cell formation and inflammation. Additionally, our present findings revealed that TBE slowed LDL oxidation *in vitro*. These data suggest that TBE can inhibit the generation and uptake of oxLDL.

MMPs released from macrophages participate in unstable plaque rupture, and high amounts of MMPs are found in macrophage-rich lesions of atherosclerotic plaque [10]. The present study’s results demonstrated that TBE inhibited the secretion of MMP-9 in THP-1 macrophages. MMP-9 is synthesized as a pro-form containing a pre-domain that is removed during activation. Further, modified LDL and pro-inflammatory cytokines are known to stimulate the expression and activation of MMP-9 [41,42]. Our results indicate that TBE may reduce matrix degradation, which could improve lesion stability.

ROS have been shown to play a well-established role in the inflammatory response. In an earlier study, the mRNA expression of pro-inflammatory cytokines in macrophages was inhibited by a ROS inhibitor, suggesting an important role for ROS in inflammatory mediator production [43]. Our present findings showed that TBE decreased the ROS production in THP-1 macrophages. ROS are generated from several sources, such as NADPH oxidase, mitochondrial respiration, and the metabolism of arachidonic acid [44]. To induce monocyte-to-macrophage differentiation, we stimulated THP-1 cells with PMA, which activates NADPH oxidase mediated by protein kinase C (PKC) [45]. It is unclear whether TBE suppressed ROS production by inhibiting the enzymes involved in ROS production or by scavenging ROS. However, the antioxidant activity of TBE is likely to account at least in part for the decrease in ROS production.

ROS have been known to activate multiple signaling pathways and transcription factors, including MAP kinases (MAPKs), PI3K/Akt, PKC, and nuclear factor-κB (NF-κB), all of which mediate inflammatory gene transcription [13,46]. It was reported that the inhibition of NADPH oxidase activity and ROS generation reduced MAPKs phosphorylation and the subsequent inflammatory mediator expression [43,47]. A genetic deficiency of NADPH oxidase diminished serum inflammatory markers and atherosclerotic lesion formation in mice [48,49]. Several polyphenols (*i.e.*, gallic acid, resveratrol, and epigallocatechin) were shown to inhibit inflammatory mediator expression via the suppression of ROS/MAPKs/NF-κB pathway [18,50], supporting our hypothesis that TBE might inhibit the expression of TNF-α, IL-1β, LOX-1, and MMP-9 due partly to its antioxidant activity.

## 5. Conclusions

In conclusion, TBE exerted an inhibitory effect on LDL oxidation and inhibited pro-inflammatory cytokines and LOX-1 expression, MMP-9 secretion, and ROS production in macrophages. Our results suggest that TBE may be effective in the reduction of atherosclerosis risk factors, but more research is needed to clarify the molecular mechanisms and functions of TBE.

## Figures and Tables

**Table 1:** Primer sequences used for the analysis of *TNF*, *IL1B*, *MSR1*, *CD36*, *OLR1* and *GAPDH*.

Gene	Sequence (5′ to 3′)
*TNF*	Forward: TGGAGAAGGGTGACCGACTC
	Reverse: TCCTCACAGGGCAATGATCC
*IL1B*	Forward: CTGTACGATCACTGAACTGC
	Reverse: CACCACTTGTTGCTCCATACT
*MSR1*	Forward: AGGCCCTCTTAAGATCAGG
	Reverse: ACAACACGGGAACCAAAGTC
*CD36*	Forward: CAATTAAAAAGCAAGTTGTCCTCGA
	Reverse: ATCACTTCCTGTGGATTTTGCA
*OLR1*	Forward: ACAGATCTCAGCCCGGCAACAAGCA
	Reverse: GGGAGACAGCGCCTCGGACTCTAAAT
*GAPDH*	Forward: TGCACCACCAACTGCTTAGC
	Reverse: GGCATGGACTGTGGTCATGAG
